# Inducible Nitric Oxide Synthase (iNOS) Is a Novel Negative Regulator of Hematopoietic Stem/Progenitor Cell Trafficking

**DOI:** 10.1007/s12015-016-9693-1

**Published:** 2016-10-17

**Authors:** Mateusz Adamiak, Ahmed Abdelbaset-Ismail, Joseph B. Moore, J Zhao, Ahmed Abdel-Latif, Marcin Wysoczynski, Mariusz Z. Ratajczak

**Affiliations:** 1 Stem Cell Institute at James Graham Brown Cancer Center, University of Louisville, 500 S. Floyd Street Rm. 107, Louisville, KY 40202 USA; 2 Institute of Molecular Cardiology, University of Louisville, Louisville, KY USA; 3 Division of Cardiovascular Medicine, Gill Heart Institute, University of Kentucky, Lexington, KY USA; 4 Department of Regenerative Medicine, Warsaw Medical University, Warsaw, Poland

**Keywords:** iNOS, NO, Stem cell mobilization, Stem cell homing, Complement cascade, HO-1

## Abstract

Nitric oxide (NO) is a gaseous free radical molecule involved in several biological processes related to inflammation, tissue damage, and infections. Based on reports that NO inhibits migration of granulocytes and monocytes, we became interested in the role of inducible NO synthetase (iNOS) in pharmacological mobilization of hematopoietic stem/progenitor cells (HSPCs) from bone marrow (BM) into peripheral blood (PB). To address the role of NO in HSPC trafficking, we upregulated or downregulated iNOS expression in hematopoietic cell lines. Next, we performed mobilization studies in iNOS^−/−^ mice and evaluated engraftment of iNOS^−/−^ HSPCs in wild type (control) animals. Our results indicate that iNOS is a novel negative regulator of hematopoietic cell migration and prevents egress of HSPCs into PB during mobilization. At the molecular level, downregulation of iNOS resulted in downregulation of heme oxygenase 1 (HO-1), and, conversely, upregulation of iNOS enhanced HO-1 activity. Since HO-1 is a negative regulator of cell migration, the inhibitory effects of iNOS identified by us can be at least partially explained by its enhancing the HO-1 level in BM cells.

**Electronic supplementary material:**

The online version of this article (doi:10.1007/s12015-016-9693-1) contains supplementary material, which is available to authorized users.

## Introduction

Evidence has accumulated that mobilization of hematopoietic stem/progenitor cells (HSPCs) from bone marrow (BM) into peripheral blood (PB) is triggered by activation of the complement cascade (ComC) [1–4]. In our previous work we demonstrated that this process, which occurs in response to certain pharmacological drugs, such as granulocyte colony stimulating factor (G-CSF) or the CXCR4 antagonist (AMD3100), is inhibited by heme oxygenase 1 (HO-1) activity [5, 6]. HO-1 metabolizes heme into biliverdin, CO, and iron, and the first two metabolites have anti-inflammatory properties, including inhibition of the ComC [7–9]. Consistent with an inhibitory effect of HO-1 on mobilization of HSPCs, HO-1^−/−^ mice are easy mobilizers [6], and downregulation of HO-1 activity in HSPCs enhances their homing after transplantation [5].

Inducible nitric oxide synthase (iNOS, also known as NOS2), like HO-1, is upregulated in response to inflammation, tissue injury, and infections and generates nitric oxide (NO), which is a gaseous free radical molecule [10, 11]. Overall, NO is also synthesized by two other nitric oxide synthase isoforms, neuronal NOS (nNOS, also known as NOS1) and endothelial NOS (eNOS, also known as NOS3), which are constitutively expressed and calcium dependent [11–14]. In contrast to nNOS and eNOS, iNOS is calcium independent and produces more NO than the other isoforms [11, 14]. Interestingly, the ComC upregulates iNOS, while NO regulates blood flow by stimulating vascular relaxation. It is also involved in neurotransmission and immune-response facilitation and exerts anti-thrombotic and anti-inflammatory effects [10, 15–18].

Interestingly, iNOS activity is enhanced during ComC activation [16], which is a crucial element of innate immunity, and its cleavage fragments, C3 and C5 (giving rise to C3a and C5a, respectively), orchestrate the egress of HSPCs from BM into peripheral blood (PB) and their homing after transplantation [1–4, 19]. However, these pro-mobilization and pro-homing effects of C3a and C5a are not related directly to the chemoattraction of HSPCs but rather indirectly to i) the induction of a proteolytic and lipolytic microenvironment in BM, ii) the chemoattraction of granulocytes and monocytes that pave the way for HSPCs to cross the BM–sinusoid endothelium barrier, and iii) increasing the level of chemotactic factors in blood plasma, including sphigosine-1-phosphate (S1P) and ceramide-1-phosphate (C1P) [2, 20–28]. In addition to the ComC, iNOS is also induced in response to several inflammatory cytokines (TNF-α, IL-1β, IFN-γ), lipopolysaccharide (LPS) endotoxin, hypoxia, and oxidative stress [10, 29].

Mobilization studies in eNOS^−/−^ mice revealed that eNOS constitutively expressed in endothelium is required for optimal vascular endothelial growth factor (VEGF)-induced mobilization of endothelial progenitor cells (EPCs) and protection from mortality after myelosuppression [30]. However, no mobilization studies have been performed so far to assess the role of inducible hematopoietic cell-expressed iNOS in the mobilization of HSPCs.

Based on the foregoing, we became interested in the potential role of iNOS in the mobilization of HSPCs. This interest was further motivated by two considerations. First, it has been reported that NO inhibits migration of granulocytes and monocytes, which, as mentioned above, are crucial in facilitating egress of HSPCs across the BM–PB barrier [11, 15, 31, 32]. Second, NO, which promotes inflammation, has also some anti-inflammatory activity limiting immune response [11, 15], and inflammation is one of the driving forces of HSPC mobilization.

To fill in this knowledge gap, we upregulated or downregulated iNOS expression in hematopoietic cells. Next, we performed mobilization studies in iNOS^−/−^ mice and, in parallel, evaluated the engraftment of iNOS^−/−^ HSPCs in wild type control animals. Since activation of the ComC is negatively regulated by heme oxygenase 1 (HO-1) [6, 33], and the ComC downregulates HO-1 [33], we tested the involvement of HO-1 in iNOS-mediated cell trafficking.

We report here for a first time that activation of iNOS negatively regulates hematopoietic cell migration and prevents egress of HSPCs into PB during mobilization. Moreover, our studies in employing hematopoietic chimeras revealed that this iNOS effect depends on its expression in hematopoietic cells.

## Materials and Methods

### Animals

In our experiments we employed female C57BL/6 J wild-type (WT) and B6.129P2-Nos2tm1Lau/J (iNOS^−/−^) 6–8-week-old mice purchased from The Jackson Laboratory (Bar Harbor, ME, USA). Animal studies were approved by the Animal Care and Use Committee of the University of Louisville (Louisville, KY, USA).

### Murine Bone Marrow-Derived Mononuclear Cells

BMMNCs were obtained by flushing femurs and tibias of pathogen-free WT or iNOS^−/−^ female mice. Cells were lysed with BD Pharm Lyse buffer (BD Biosciences, San Jose, CA, USA) to remove red blood cells (RBCs), washed, and resuspended in appropriate media for further experiments. When necessary, murine Sca-1^+^ cells were isolated as described [21].

### Cell Lines

HEL and K562 cell lines were propagated in RPMI 1640 medium (Life Technologies), supplemented with 10 % FBS (Seradigm), 1× GlutaMAX™ (Life Technologies), and 1× penicillin–streptomycin (Life Technologies). All cell lines were passaged every 2–3 days to maintain concentrations in the approximate range of 200,000–800,000 cells per ml.

### Human NOS2 Overexpression and shRNA-Mediated Knockdown

Lentiviral particles utilized in the establishment of NOS2-overexpressing or NOS2-knockdown cell lines were derived using the ViraPower™Lentiviral Expression System (Life Technologies) according to the manufacturer’s protocol. Subsequently, HEL or K562 suspension cells were transduced via spinoculation. Briefly, cells (2 × 10^5^ in 2 ml of complete medium) were mixed with 2 ml of viral supernatant and polybrene (8 μg/ml final concentration). The cell–virus suspension was then centrifuged at 800 x g for 2 h at 37 °C. At the conclusion of spinoculation, the viral medium was aspirated, and the cells were resuspended in complete medium and then incubated in a 37 °C incubator (5 % CO_2_). Seventy-two hours post viral transduction, the cells underwent antibiotic-mediated selection using puromycin (1 μg/ml). All cell lines were maintained under antibiotic selection pressure throughout the duration of their expansion prior to experimentation. A detailed list of the lentiviral vectors employed is found in Table 1.

**Table 1:** NOS2 overexpression and shRNA-knockdown viral vectors

Human NOS2 overexpression	Vector construct	Company
Control vector (empty vector)	pLenti6-MCS-GFP-2 A-Puro	N/A
Human NOS2 vector (Accession BC130283)	pLenti-GIII-CMV-Human NOS2-GFP-2 A-Puro	abm® inc.
Human NOS2 shRNA knockdown	Vector construct	Company
Control shRNA (non-target control shRNA)	pLenti6-U6-NT shRNA-GFP-2 A-Puro2	N/A
NOS2 Mission® TRC shRNA (shRNA 1)	Mission shNOS2 (TRCN0000231570)	Sigma-Aldrich®
NOS2 Mission® TRC shRNA (shRNA 2)	Mission shNOS2 (TRCN0000231573)	Sigma-Aldrich®

### Mobilization Studies

WT and iNOS^−/−^ mice were injected subcutaneously (s.c.) with 100 μg/kg G-CSF (Amgen, Thousand Oaks, CA, USA) daily for 3 (short mobilization) or 6 (long mobilization) days or one dose of AMD3100 (Sigma-Aldrich, 5 mg/kg, intraperitoneally). At 6 h after the last G-CSF administration or at 1 h after AMD3100 injection, the mice were bled from the retro-orbital plexus for hematology analysis, and peripheral blood (PB) was obtained from the vena cava with a 25-gauge needle and 1-ml syringe containing 50 μl of 100 mM EDTA. Mononuclear cells (MNCs) were obtained by hypotonic lysis of RBCs in BD Pharm Lyse buffer (BD Biosciences).

### Fluorescence-Activated Cell Sorting (FACS) Analysis

The following monoclonal antibodies were used to perform staining of Lin^−^/Sca-1^+^/c-Kit^+^ (SKL) cells and Lin^−^/Sca-1^+^/CD45^+^ (hematopoietic stem cells [HSCs]): FITC–anti-CD117 (also known as c-Kit, clone 2B8; BioLegend, San Diego, CA, USA) and PE–Cy5–anti-mouse Ly-6 A/E (also known as Sca-1, clone D7; eBioscience, San Diego, CA, USA). All anti-mouse lineage markers, (Lin) anti-CD45R/B220 (clone RA3-6B2), anti-Ter-119 (clone TER-119), anti-CD11b (clone M1/70), anti-T cell receptor β (clone H57–597), anti-Gr-1 (clone RB6-8C5), anti-TCRγδ (clone GL3), and anti-CD45 (clone 30-F11), were purchased from BD Biosciences and conjugated with PE as described. Staining was performed in RPMI 1640 medium containing 2 % FBS. All monoclonal antibodies (mAbs) were added at saturating concentrations, and the cells were incubated for 30 min on ice, washed twice, and analyzed with an LSR II flow cytometer (BD Biosciences).

### Evaluation of HSPC Mobilization

For evaluation of circulating colony-forming unit-granulocyte/macrophage (CFU-GM) and SKL cells the following formula was used: (number of white blood cells [WBCs]) x number of CFU-GM colonies)/number of WBCs plated = number of CFU-GM per μl of PB; and (number of WBCs x number of SKL cells)/number of gated WBCs = number of SKL cells per μl of PB.

### Short-Term Homing Experiments

Lethally irradiated WT mice (γ-irradiation at 1000 cGy) 24 h after irradiation were transplanted (by tail vein injection) with 3 × 10^6^ BM cells from WT or iNOS^−/−^ mice labeled with the PKH67 green fluorescent cell linker according to the manufacturer’s protocol (Sigma-Aldrich, St Louis, MO, USA). At 24 h after transplant, BM cells from the femurs were isolated via Ficoll-Paque and divided, and 30 % of the cells were analyzed by FACS. The rest of the cells were plated in serum-free methylcellulose cultures and stimulated to grow CFU-GM colonies with mGM-CSF (25 ng/ml) and mIL-3 (10 ng/ml). After 7 days of incubation (37 °C, 95 % humidity, and 5 % CO_2_), the number of colonies was scored under an inverted microscope.

### Evaluation of Engraftment

For short-term engraftment experiments, WT mice were irradiated with a 1000 cGy of γ-irradiation. After 24 h, mice were transplanted by tail vein injection with 1.5 × 10^5^ BM cells from WT or iNOS^−/−^ mice. Femora of transplanted mice were flushed with PBS on day 12 post-transplant. Purified via Ficoll-Paque, BM cells were plated in serum-free methylcellulose cultures and stimulated to grow CFU-GM colonies with mGM-CSF (25 ng/ml) and IL-3 (10 ng/ml). After 7 days of incubation (37 °C, 95 % humidity, and 5 % CO_2_) the number of colonies was scored under an inverted microscope. Spleens were also removed, fixed in Telesyniczky’s solution for CFU-S assays, and the colonies on the surface of the spleen counted.

### Recovery of Leukocytes and Platelets

WT mice were lethally irradiated, and after 24 h, the animals were transplanted by tail vein injection with 2.5 × 10^5^ BM cells from WT or iNOS^−/−^ mice. Transplanted mice were bled at various intervals from the retro-orbital plexus to obtain samples for white blood cell and platelet counts. Fifty microliters of PB was taken from the retro-orbital plexus of the mice into EDTA-coated Microvette tubes (Sarstedt Inc., Newton, NC, USA) and run within 2 h of collection on a HemaVet 950FS hematology analyzer (Drew Scientific Inc., Oxford, CT, USA).

### Western Blotting

K562-pCMV6-hiNOS, HEL-pCMV6-hiNOS, K562-shiNOS, HEL-shiNOS and their respective control cells were harvested, centrifuged, and washed with cold PBS. For protein extraction, the cells were treated with cold RIPA lysis buffer, supplemented with protease and phosphatase inhibitors (Santa Cruz Biotech), for 30 min on ice and centrifuged at 15,000 rpm at −4 °C for 15 min. The total protein concentration per sample was measured using the Pierce BCA Protein Assay Kit (Pierce, Rockford, IL) and Multimode Analysis Software (Beckman Coulter). The concentration-adjusted extracted proteins (50 μg/each sample) were then separated on a 4–12 % SDS-PAGE gel and transferred to a PVDF membrane (Bio-Rad). All membranes were blocked with 2.5 % nonfat dry milk in Tris-buffered saline containing 0.1 % Tween (TBST) for 1 h at room temperature. After washing with TBST, the membranes were incubated with rabbit anti-HO-1 polyclonal antibody (Enzo Life Sciences, NY, USA; diluted 1:1000) overnight at 4 °C. To assure equal protein loading in each lane, the blots were then reprobed with a rabbit anti-β-actin monoclonal antibody (Novus Biologicals, USA; diluted 1:1000). All membranes were then treated with enhanced chemiluminescence (ECL) reagent (Amersham Life Sciences) and subsequently exposed to film (Hyperfilm, Amersham Life Sciences). For protein band visualization, an automatic film developer supplied with fresh warm developer and fixer solutions was used. The intensities of the blots obtained were quantified using ImageJ software (National Institutes of Health, Bethesda, MD, USA).

### Real-Time Quantitative Reverse-Transcription PCR of Cell Lines

Total RNA was isolated with the RNeasy Kit (Qiagen, Valencia, CA, USA) from hematopoietic cells in which iNOS was up- and downregulated and their respective control cells. Expression of iNOS at the mRNA level was also evaluated in hematopoietic cell lines in which HO-1 was up- and downregulated in comparison with control cell lines. The RNA was reverse-transcribed with MultiScribe reverse transcriptase and oligo-dT primers (Applied Biosystems, Foster City, CA, USA). Quantitative assessment of mRNA levels was done by real-time RT-PCR using an ABI 7500 instrument with Power SYBR Green PCR Master Mix reagent. PCR conditions were as follows: 95 °C (15 s), 40 cycles at 95 °C (15 s), and 60 °C (1 min). According to melting point analysis, only one PCR product was amplified under these conditions. The relative quantity of a target, normalized to the endogenous β2-microglobulin gene as control and relative to a calibrator, is expressed as 2^–DDCt^ (fold difference), where Ct is the threshold cycle, DCt = (Ct of target genes) − (Ct of the endogenous control gene, β2-microglobulin), and DDCt = (DCt of samples for the target gene) − (DCt of the calibrator for the target gene). The following primer pairs were used for analysis of iNOS expression: 5-CAG CGG GAT GAC TTT CCA A-3 (forward) and 5- AGG CAA GAT TTG GAC CTG CA-3 (reverse). For HO-1 expression: 5-GGG TGA TAG AAG AGG CCA AGA CT-3 (forward) and 5-AGC TCC TGC AAC TCC TCA AGA-3 (reverse).

### Transwell Chemotaxis Assay

To render the cells quiescent, K562 and HEL cell lines were incubated overnight in RPMI 1640 medium containing 0.5 % bovine serum albumin (BSA). BMMNCs from WT and iNOS^−/−^ mice were made quiescent for 3 h in the same medium, and some were incubated for 1 h with different doses of the iNOS inhibitor, N^*6*^-(1-Iminoethyl)-L-lysine hydrochloride (L-NIL; Tocris Bioscience, Bristol, UK). Subsequently, cells were washed and resuspended in assay medium (RPMI 1640 with 0.5 % BSA). Cells were aliquoted at a density of 1 × 10^5^ cells in 100 μl with an 8-μm syringe (for human cell lines) and 1 × 10^6^ cells in 100 μl with a 5-μm syringe (for murine BMMNCs) into polycarbonate membrane inserts in a Costar Transwell 24-well plate (Costar Corning, Cambridge, MA, USA) and incubated for 3 h of chemotaxis at 37 °*C. medium* with 0.5 % BSA (650 μl/well) containing no chemoattractant (negative control), stromal-derived factor 1 (SDF-1, 50 ng/ml), sphingosine-1-phosphate (S1P, 0.1 μM), ceramide-1-phosphate (C1P, 100 μM), or adenosine triphosphate (ATP, 0.5 μg/ml) was added to the lower chambers of the plate. After 3 h of incubation, the cells from the lower chambers were collected. The number of human cell lines and murine BM-derived cells were scored by FACS (Becton Dickinson, Franklin Lakes, NJ, USA). Briefly, the cells were gated according to their forward scatter (FSC) and side scatter (SSC) parameters and counted during a 30-s acquisition at a high flow rate. After chemotaxis from the lower chamber, the murine cells were resuspended in human methylcellulose base medium provided by the manufacturer (R&D Systems, Minneapolis, MN, USA), supplemented with murine and human granulocyte/macrophage colony stimulating factor (GM-CSF, 25 ng/ml) and interleukin-3 (IL-3, 10 ng/ml) for determining the number of CFU-GM colonies. Cultures were incubated for 7 days (37 °C, 95 % humidity, and 5 % CO_2_), at which time they were scored under an inverted microscope for the number of colonies.

### Fibronectin Adhesion Assay

Human cell lines and murine BMMNCs at a density of 5 × 10^4^/100 μl were made quiescent overnight or for 3 h, respectively, and some were next incubated with different doses of L-NIL for 1 h. Subsequently cells were washed by centrifugation and resuspended in RPMI-1640 medium. Cell suspensions were added directly to 96-well plates that had been coated before the experiment with fibronectin (10 μg/ml), incubated overnight at 4 °C, and then blocked with medium containing 0.5 % BSA for 2 h. After 15 min at 37 °C, the non-adherent cells were then washed from the wells, and all adherent cells were counted using an inverted microscope.

### Measurement of Intracellular Nitric Oxide (NO)

K562-pCMV6-hiNOS, HEL-pCMV6-hiNOS, K562-shiNOS, HEL-shiNOS, RAJI-pCMV6-hHO-1, RAJI-shHO-1, and their respective control cell lines were centrifuged and suspended in their culture medium in poly-D-lysine-coated wells (15 × 10^4^ cells/well) of 96-well plates. Each cell line was individually evaluated for NO levels using the Cell Meter™ Orange Fluorimetric Intracellular Nitric Oxide Assay Kit (AAT Bioquest, #16,350). The loaded plates were centrifuged at 800 rpm for 2 min. Next, cells were incubated with Nitrixyte™ Orange probe working solution for 30 min at 37 °C to detect free NO in the cells. After assay buffer II was added, the orange fluorescence signals were then measured using a microplate reader at an excitation wavelength of 540 nm and an emission wavelength of 590 nm (cut off at 570 nm) in bottom-read mode.

### Statistical Analysis

All results are presented as mean ± SD. Statistical analysis of the data was done using Student’s *t-*test for unpaired samples (Excel, Microsoft Corp., Redmond, WA, USA) with a value of *p* ≤ 0.05 considered significant.

## Results

### Upregulation of iNOS in Established Hematopoietic Cell Lines Impairs their Chemotactic Response to SDF-1 and S1P Gradients and Enhances Cell Adhesion

**Fig. 1:**
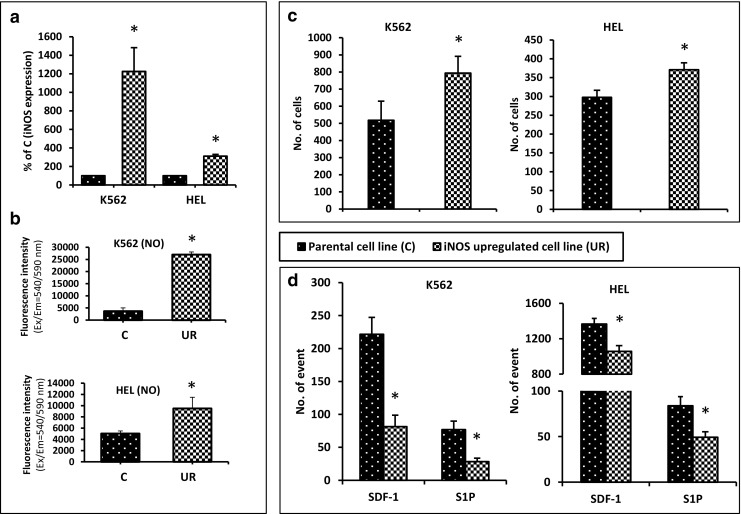
Influence of iNOS upregulation on chemotaxis and adhesion of human hematopoietic cell lines (K562 and HEL). Panel A. iNOS expression was evaluated at the mRNA level by real-time PCR. Results from three independent experiments are pooled together. Panel B. Measurement of NO levels in the tested cells lines. *p ≤ 0.005. Panel C. Fibronectin adhesion assay. The number of adherent cells is indicated, and results from three separate experiments are pooled together. *p ≤ 0.01. Panel D. The chemotactic responsiveness of iNOS-upregulated cells to SDF-1 or S1P gradients compared with the migration of control parental cells. Results are combined from three independent experiments. *p ≤ 0.05

To address the effect of iNOS on migration and adhesion of hematopoietic cells, we established two human hematopoietic cell lines in which iNOS had been overexpressed after transducing cells with an iNOS-encoding vector. Figure 1A shows real time RT-PCR results in which iNOS was upregulated in HEL and K562 cell lines, and these cells expressed free NO at higher levels (Fig. 1B). Moreover, in functional assays iNOS overexpression was correlated with enhanced adhesion of cells to fibronectin-coated plates (Fig. 1C) and, more importantly, had reduced migration in response to SDF-1 and S1P gradients (Fig. 1D).

### Downregulation of iNOS in Established Hematopoietic Cell Lines Increases their Chemotactic Response to SDF-1 and S1P Gradients and Impairs Cell Adhesion

**Fig. 2:**
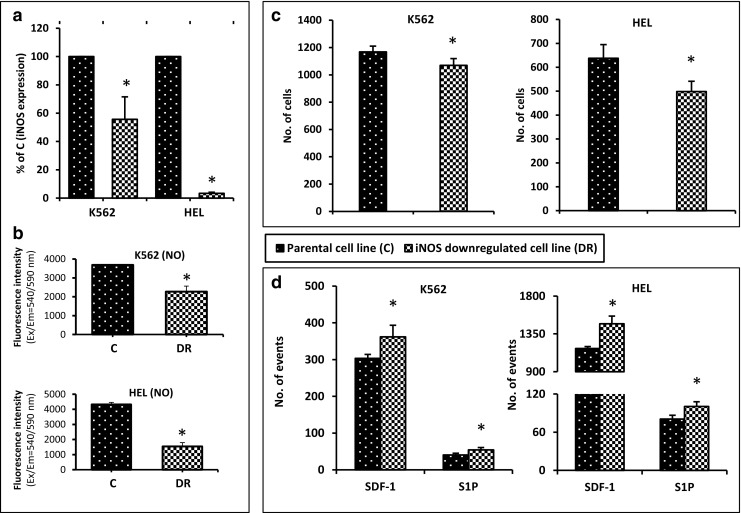
Influence of iNOS downregulation on chemotaxis and adhesion of human hematopoietic cell lines (K562 and HEL). Panel A. iNOS expression was evaluated at the mRNA level by real-time PCR. Results from three independent experiments are pooled together. Panel B. Measurement of NO levels in the tested cell lines. *p ≤ 0.005. Panel C. Fibronectin adhesion assay. The number of adherent cells is indicated, and results from three separate experiments are pooled together. *p ≤ 0.01. Panel D. The chemotactic responsiveness of iNOS-downregulated cells to SDF-1 or S1P gradients compared with migration of control parental cells. Results are combined from three independent experiments. *p ≤ 0.05

Next, we successfully downregulated iNOS expression in the same HEL and K562 cells by employing a shRNA strategy. Figure 2A shows real-time RT-PCR results for iNOS mRNA expression, and Fig. 2B shows the level of free NO in these cells. We found that downregulation of iNOS correlated with decreased adhesion to fibronectin-coated plates (Fig. 2C) and enhanced migration in response to SDF-1 and S1P gradients (Fig. 2D).

### iNOS-Deficient Mice Are easily Mobilized by G-CSF and AMD3100

Based on the above observations that iNOS modulates cell migration, we moved to a murine model of iNOS deficiency and employed iNOS^−/−^ animals for in vivo studies. Supplementary Fig. 1 shows that the iNOS-deficient mice employed in our studies have normal PB cell counts (Supplementary Fig. 1A), red blood cell parameters (Supplementary Fig. 1B), and numbers of bone marrow-residing HSPCs (Supplementary Fig. 1C) and clonogenic progenitors (Supplementary Fig. 1D) compared with WT animals under steady-state conditions.

**Fig. 3:**
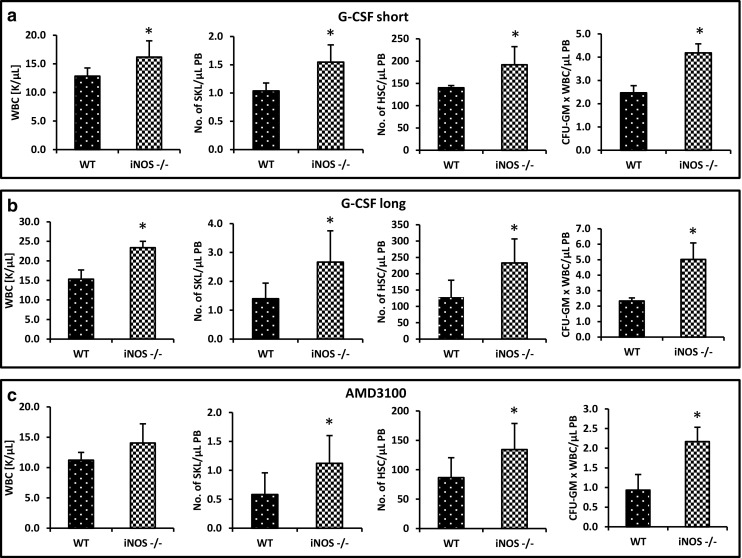
iNOS^−/−^ mice are easily mobilized with G-CSF and AMD3100. MNCs were isolated from WT and iNOS^−/−^ mice after 3-day (panel A) and 6-day (panel B) injections of G-CSF and a single dose of AMD3100 (panel C). Animals were killed 6 h after the last G-CSF injection and 1 h after AMD3100 mobilization, and the numbers of WBCs, SKL (Sca-1^+^ c-kit^+^ Lin^−^) cells, HSCs (Sca-1^+^ CD45^+^ Lin^−^), and CFU-GM clonogenic progenitors from PB were evaluated. Results from two separate experiments are pooled together. *p ≤ 0.05

**Fig. 4:**
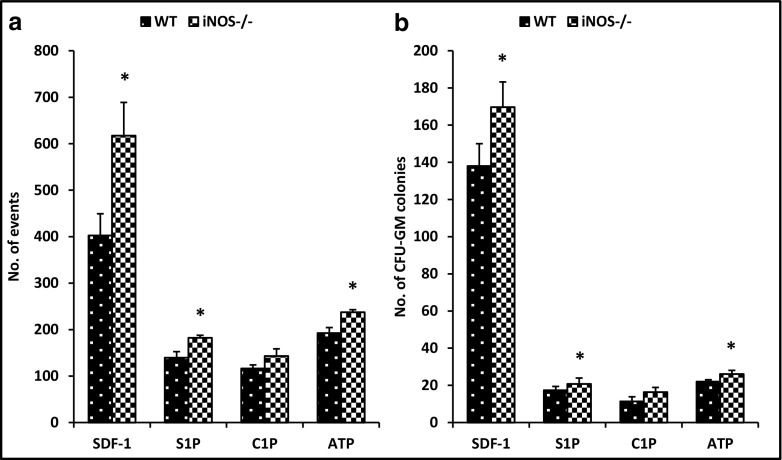
Impact of iNOS deficiency on the chemotactic activity of BMMNCs. The chemotactic responsiveness of WT and iNOS^−/−^-derived BMMNCs to SDF-1, S1P, C1P, and ATP gradients evaluated by FACS (*panel A*) and clonogenic CFU-GM progenitors (*panel B*). Results are combined from three independent experiments.*p ≤ 0.05

Next, we performed in vivo mobilization experiments. Figure 3 shows that iNOS^−/−^ mice display enhanced 3-day (short) G-CSF-induced (Fig. 3A), 6-day (long) G-CSF-induced (Fig. 3B), and AMD3100-induced mobilization (Fig. 3C). This result indicates that these mice are easily mobilized and that iNOS may exert negative effects on the egress of HSPCs from BM into PB. These results correspond with an enhanced responsiveness of BMMNCs (Fig. 4A) and clonogeneic CFU-GMs (Fig. 4B) to HSPC chemoattractants, including SDF-1, S1P, C1P, and ATP.

### iNOS^−/−^ BMMNCs Show Enhanced Homing and Engraftment in WT Animals

**Fig. 5:**
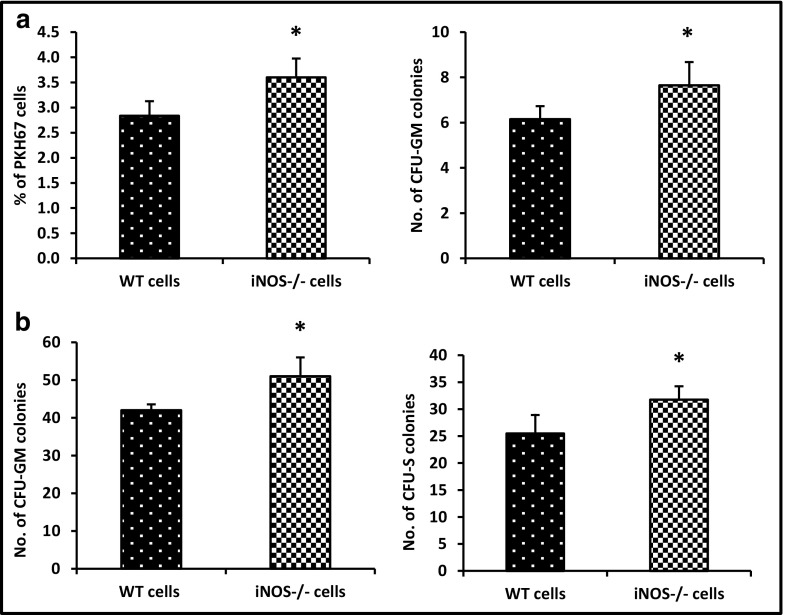
Increase in homing and short-term engraftment of HSPCs from iNOS^−/−^ mouse BM. Panel A. Lethally irradiated WT mice (six mice per group) were transplanted with 5 × 10^6^ WT or iNOS^−/−^ bone marrow mononuclear cells (BMMNCs) labeled with PKH67; 24 h after transplantation femoral BMMNCs were harvested; the number of labeled cells in murine BM was evaluated by FACS (*panel A, left*); and the number of clonogenic CFU-GM progenitors was enumerated in an in vitro colony assay (*panel A, right*). Panel B. Lethally irradiated WT mice (six mice per group) were transplanted with 1.5 × 10^5^ BMMNCs from WT or iNOS^−/−^ mice, and 12 days after transplantation femoral BMMNCs were harvested and plated to count the number of CFU-GM colonies (*panel B, left*), and spleens were removed for counting the number of CFU-S colonies (*panel B, right*). No colonies were formed in lethally irradiated and untransplanted mice (irradiation control). The data in panels A and B represent the combined results from two independent experiments (*n* = 10). *p < 0.005

Next, we performed homing studies employing PKH67-labeled iNOS^−/−^ and WT control mouse-derived BMMNCs transplanted into lethally irradiated WT animals. Twenty-four hours after transplantation, we observed an increased number of transplanted PKH67^+^ cells from iNOS-deficient mice as well as an increased number of clonogenic progenitors that were able to grow CFU-GM colonies after isolation from BM and plating in methylcellulose cultures (Fig. 5A).

In parallel, we sacrificed some of the animals on day 12 after transplantation and evaluated the number of colony-forming units in spleen (CFU-S) and the content of clonogenic CFU-GM progenitors in BM (Fig. 5B). Again, we observed a statistically significant beneficial effect of iNOS deficiency on short-term engraftment. Thus, both statistically significant enhanced homing and short-term engraftment of iNOS-deficient BM cells are correlated with enhanced migration of these cells in response to hematopoietic chemoattractant gradients, as shown in Fig. 4.

**Fig. 6:**
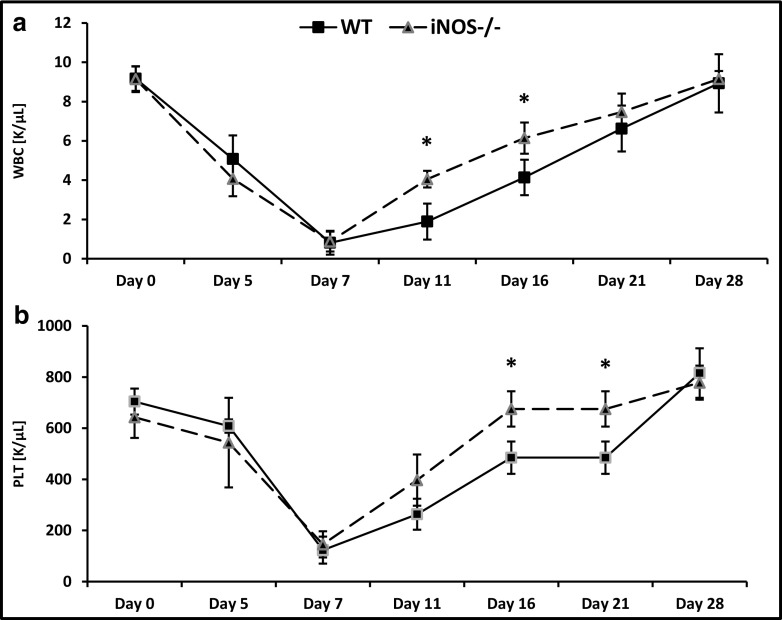
Accelerated engraftment of iNOS^−/−^ BMMNCs. Lethally irradiated WT mice were transplanted with 1 × 10^6^ BMMNCs from WT or iNOS^−/−^ cells. White blood cells (*panel A*) and platelets (*panel B*) were counted at intervals (0, 5, 7, 11, 16, 21, and 28 days after transplantation). Results are combined from two independent experiments (six mice per group, *n* = 12). *p < 0.05

Finally, we evaluated the short term engraftment of BM-MNCs from iNOS^−/−^mice by measuring the kinetics of leukocyte (Fig. 6A) and platelet (Fig. 6B) recovery in these animals. We found that mice transplanted with iNOS^−/−^ BMMNCs had a significantly accelerated recovery of leukocyte and platelet counts in peripheral blood.

### Downregulation of iNOS in Murine BMMNCs by L-NIL, a Small-Molecule Inhibitor that Increases their Migration in Response to SDF-1 and S1P Gradients

**Fig. 7:**
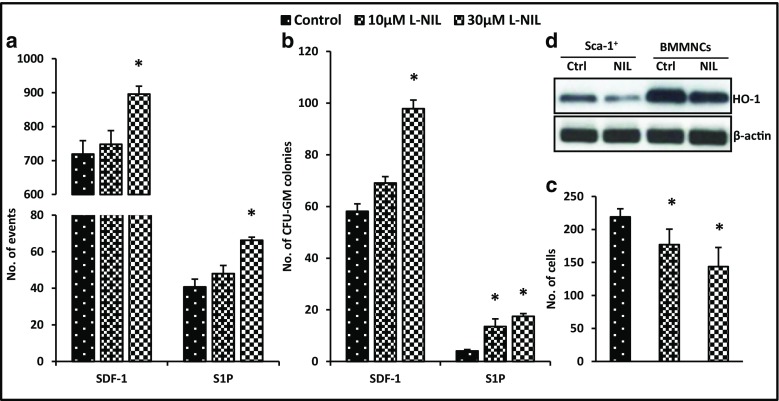
The influence of an iNOS inhibitor (L-NIL) on chemotaxis and adhesion of murine BM-MNCs. The chemotactic responsiveness to SDF-1 and S1P gradients of murine BMMNCs (evaluated by FACS) (*Panel A*) and clonogenic CFU-GM progenitors (*Panel B*) exposed to two different dosages of iNOS inhibitor. Results are combined from three independent experiments.*p ≤ 0.05. Panel C. The effect of iNOS inhibition on adhesion of murine BMMNCs to fibronectin. Data from four separate experiments are pooled together. *p ≤ 0.01. Panel D. Detection of the HO-1 protein expression level by western blot in WT Sca-1^+^ cells and BMMNCs exposed to high doses of L-NIL. The same membranes were reprobed with β-actin to confirm equal loading of total protein

Based on encouraging results with iNOS-downregulated cell lines and homing/engraftment studies with iNOS^−/−^ BMMNCs, we exposed murine BMMNCs to nontoxic doses of the small-molecule iNOS inhibitor L-NIL (Supplementary Fig. 2) and evaluated the responsiveness of these cells to SDF-1 and S1P homing gradients. Again, inhibition of iNOS in murine BMMNCs resulted in enhanced migration (Fig. 7A, B) and decreased adhesion to fibronectin-coated plates (Fig. 7C). These results indicate that iNOS expression can be successfully modulated by small-molecule inhibitors and potentially employed to accelerate the homing of HSPCs. More importantly, we found that exposure of murine Sca-1^+^ cells and BMMNCs to L-NIL resulted in downregulation of HO-1 (Fig. 7D).

### HO-1 Is Upregulated in Hematopoietic Cells with iNOS Overexpression and Expressed at Lower Levels in Cell Lines with iNOS Downregulation

**Fig. 8:**
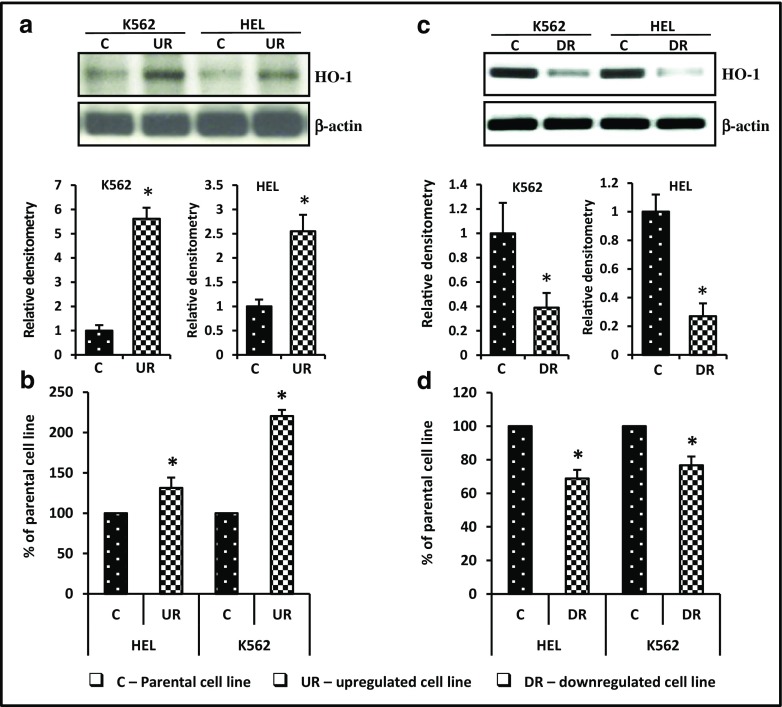
Correlation between iNOS up- and downregulation and HO-1 expression in experimental hematopoietic cell lines. Panels A and C. Expression of HO-1 following iNOS upregulation (*Panel A*) or downregulation (*Panel C*) was evaluated at the protein level by western blotting, and representative blots and their densitometry analysis are shown (*upper and lower panels*, respectively). The same membranes were reprobed with β-actin to confirm equal loading of total protein. Panel B and D. In parallel, HO-1 expression in the cells employed was assessed at the mRNA level by real-time PCR. Results from three independent experiments are pooled together. *p ≤ 0.005

We have previously reported that HO-1 is a negative regulator of cell trafficking and an inhibitor of ComC activation [6, 33]. Therefore, we became interested in the potential relationship between expression of iNOS and HO-1 activity. As shown in Fig. 8, upregulation of iNOS in HEL and K-562 cells leads to an increase in HO-1 expression at the protein (Fig. 8A) and mRNA levels (Fig. 8B). By contrast, downregulation of iNOS in these cells correlates with lower expression of HO-1 at the protein (Fig. 8C) and mRNA levels (Fig. 8D).

Finally, to address whether, vice versa, HO-1 overexpression affects iNOS expression, we employed the RAJI cell line, in which we overexpressed or downregulated HO-1 [5] and evaluated the iNOS mRNA level by real-time PCR (Supplementary Fig. 3A, C) and, in parallel, the NO levels in these cells (Supplementary Fig. 3B, D). We observed a positive correlation between HO-1 and iNOS expression.

## Discussion

The most important observation of this report is that iNOS is a novel negative regulator of the trafficking of HSPCs, and this negative effect on cell migration is related to its effect on HO-1 activity. Moreover, our results suggest that expression of iNOS and HO-1 is regulated in hematopoietic cells in a similar way. The inhibition of iNOS, as we reported for HO-1 [5, 6], may have practical applications in enhancing the homing of HSPCs to BM after transplantation as well as enhancing the mobilization of HSPCs from BM into PB in poor mobilizers.

In addition to hematopoietic cells, NO is also synthesized by many other cell types in the body in response to cytokines or stress situations, and its role is still not well understood. Initially, NO was considered to be mainly an antimicrobial, tumoricidal, and tissue-damaging effector molecule operating as part of the innate immune system [34–37]. However, today it is accepted that, in addition to innate immune responses, NO also strongly affects adaptive immune responses and exerts certain cytoprotective effects. It has been postulated that, in addition to pro-inflammatory effects, iNOS, and to some extent also other NOS isoforms (nNOS and eNOS), limits immune responses and has anti-inflammatory functions [11]. These include, for example, the eNOS-, iNOS-, or eNOS-dependent inhibition of leukocyte adhesion and transendothelial migration [38, 39]. NO may also exert anti-inflammatory effects by affecting polarization of macrophages due to reduced proinflammatory M1 and increased anti-inflammatory M2 phenotypic properties [15].

It is known that iNOS and HO-1 are upregulated in parallel in response to infection and tissue or organ damage [36, 40, 41]. However, the mutual relationship between these stress-induced enzymes is still not very well understood. While overexpression of HO-1 or exposure of mouse or human cells to HO-1 hemin metabolism products (CO or Fe^2+^) may suppress cytokine- or LPS-induced iNOS expression [42], on the other hand, depending on the cell system studied, NO may upregulate expression of HO-1 and its product CO [43].

We observed that in hematopoietic cells changes in expression of iNOS parallel changes in expression of HO-1. Thus, at the molecular level, one of the most plausible explanations for enhanced migration of HSPCs with downregulated iNOS could be because of the decrease of a negative regulator of cell migration, HO-1 [5, 6].

Our results also indicate that different isoforms of NOS may have different effects on cell trafficking. While mobilization studies in eNOS-deficient mice revealed that constitutive expression of eNOS in endothelium is required for mobilization of endothelial progenitor cells in response to VEGF stimulation [30], our results with hematopoietic cell-expressed iNOS indicate that this isoform has the opposite, anti-mobilization, effect on HSPCs in response to G-CSF and AMD3100. Since iNOS is also expressed in addition to constitutively expressed eNOS in endothelial cells, further studies are needed to determine whether these nitric oxide synthases affect mobilization of endothelial progenitor cells in opposite ways. Moreover, taking into consideration the broad tissue expression of nNOS, it would be interesting to perform similar studies in nNOS^−/−^ animals [44]. In justification of such studies, nestin-positive cells that regulate stem cell trafficking have been described in BM [45, 46], and these cells could be potential effector cells for the biological effects of nNOS.

While several factors that enhance mobilization of HSPCs have been identified, very little is known about the physiological inhibitors of this process. In this context, our results with HO-1 [5, 6] and with iNOS that are reported here shed new light on regulation of this process. Since excessive inflammation mediated by ComC activation contributes to various complications (e,g., sepsis), it is important to identify molecules that attenuate complement-mediated inflammation. Thus, further studies are needed to better understand the reciprocal relationship between activation of the ComC, which may initially promote expression of both of these stress-induced enzymes, and the subsequent negative feedback that limits activation of the ComC. In support of such a relationship, it is know that biliverdin and bilirubin are potent inhibitors of activation of the ComC by inhibiting its activation directly in the classical pathway of ComC activation at the C1 level by physically interacting with complement proteins [8, 47] and by modulating expression of C5aR [7]. This modulation has important implications for the mobilization process, as the C5a–C5aR interaction is crucial for promoting egress of HSPCs from BM into PB, and mice deficient in C5a or C5aR are poor mobilizers [2].

In conclusion, we have demonstrated for the first time that iNOS plays an important role in mobilization as well as engraftment of HSPCs. Further studies are needed to confirm whether modulation of iNOS in human HSPCs, as in their murine counterparts, could be employed as a novel strategy to enhance homing and accelerate engraftment of HSPCs after transplantation. This would be important, particularly in clinical situations in which the number of HSPCs to be transplanted is low (e.g., from poor-mobilizing donors or UCB transplantations) or, on the other hand, in patients that are poor mobilizers in response to classical pro-mobilizing agents.

## Electronic supplementary material

Supplementary Figure 1 Hematological parameters in iNOS^−/−^ mice. PB parameters were evaluated using a HemaVet 950FS analyzer, and iNOS^−/−^ mice had normal white blood cells (WBC), neutrophils (NE), lymphocytes (LY), and monocytes (MO) (Panel A). Compared with WT mice, iNOS^−/−^ mice had normal numbers of red blood cells (RBC), hemoglobin content (HB), hematocrit (HCT), mean volume of erythrocytes (MCV), mean content of hemoglobin (MCH), mean concentration of hemoglobin in erythrocytes (MCHC), and red cell distribution width (RDW) (Panel B). Under steady-state conditions, there were also no differences between iNOS^−/−^ and WT mice in the numbers of SKL cells and hematopoietic stem cells (HSCs) circulating in PB (Panel C). The bone marrow of WT and iNOS^−/−^ mice was also isolated and evaluated for the numbers of CFU-GM, BFU-E, and CFU-Meg clonogenic progenitors in in vitro assays, and there were also no significant differences between control and iNOS^−/−^ mice (Panel D). Data represent an average of at least eight mice tested per experimental group. *p ≤ 0.05 (PPTX 132 kb)

Supplementary Figure 2 L-NIL toxicity studies. Toxicity studies of an iNOS inhibitor (L-NIL) were performed based on evaluation of the number of CFU-GM (Panel left) and BFU-E (Panel right) clonogenic progenitors in in vitro assays. Murine bone marrow mononuclear cells were incubated with medium alone or different doses of L-NIL for 1 h and then supplemented for CFU-GM and BFU-E colonies. BM hematopoietic clonogenic progenitors were scored after 7 days of incubation, and data from two separate experiments are pooled together. (PPTX 72 kb)

Supplementary Figure 3 Expression of HO-1 directly correlates with iNOS expression in hematopoietic cell lines. Expression of iNOS in the RAJI cell line with up- and downregulation of HO-1 was analyzed by qPCR (panels A and C). In parallel, we measured the NO levels in these cells as described in the Materials and Methods section (panels B and D). *p ≤ 0.005. (PPTX 85 kb)
