# Supplementary material for: Inducible Nitric Oxide Synthase (iNOS) Is a Novel Negative Regulator of Hematopoietic Stem/Progenitor Cell Trafficking
Source: Stem Cell Rev. 2016 Oct 17;13(1):92–103. doi: 10.1007/s12015-016-9693-1 (PMC5346113; doi:10.1007/s12015-016-9693-1)
Supplement: Supplementary file 1 — Hematological parameters in iNOS−/− mice. PB parameters were evaluated using a HemaVet 950FS analyzer, and iNOS−/− mice had normal white blood cells (WBC), neutrophils (NE), lymphocytes (LY), and monocytes (MO) (Panel A). Compared with WT mice, iNOS−/− mice had normal numbers of red blood cells (RBC), hemoglobin content (HB), hematocrit (HCT), mean volume of erythrocytes (MCV), mean content of hemoglobin (MCH), mean concentration of hemoglobin in erythrocytes (MCHC), and red cell distribution width (RDW) (Panel B). Under steady-state conditions, there were also no differences between iNOS−/− and WT mice in the numbers of SKL cells and hematopoietic stem cells (HSCs) circulating in PB (Panel C). The bone marrow of WT and iNOS−/− mice was also isolated and evaluated for the numbers of CFU-GM, BFU-E, and CFU-Meg clonogenic progenitors in in vitro assays, and there were also no significant differences between control and iNOS−/− mice (Panel D). Data represent an average of at least eight mice tested per experimental group. *p ≤ 0.05 (PPTX 132 kb) [file 12015_2016_9693_MOESM1_ESM.pptx]

## Slide 1
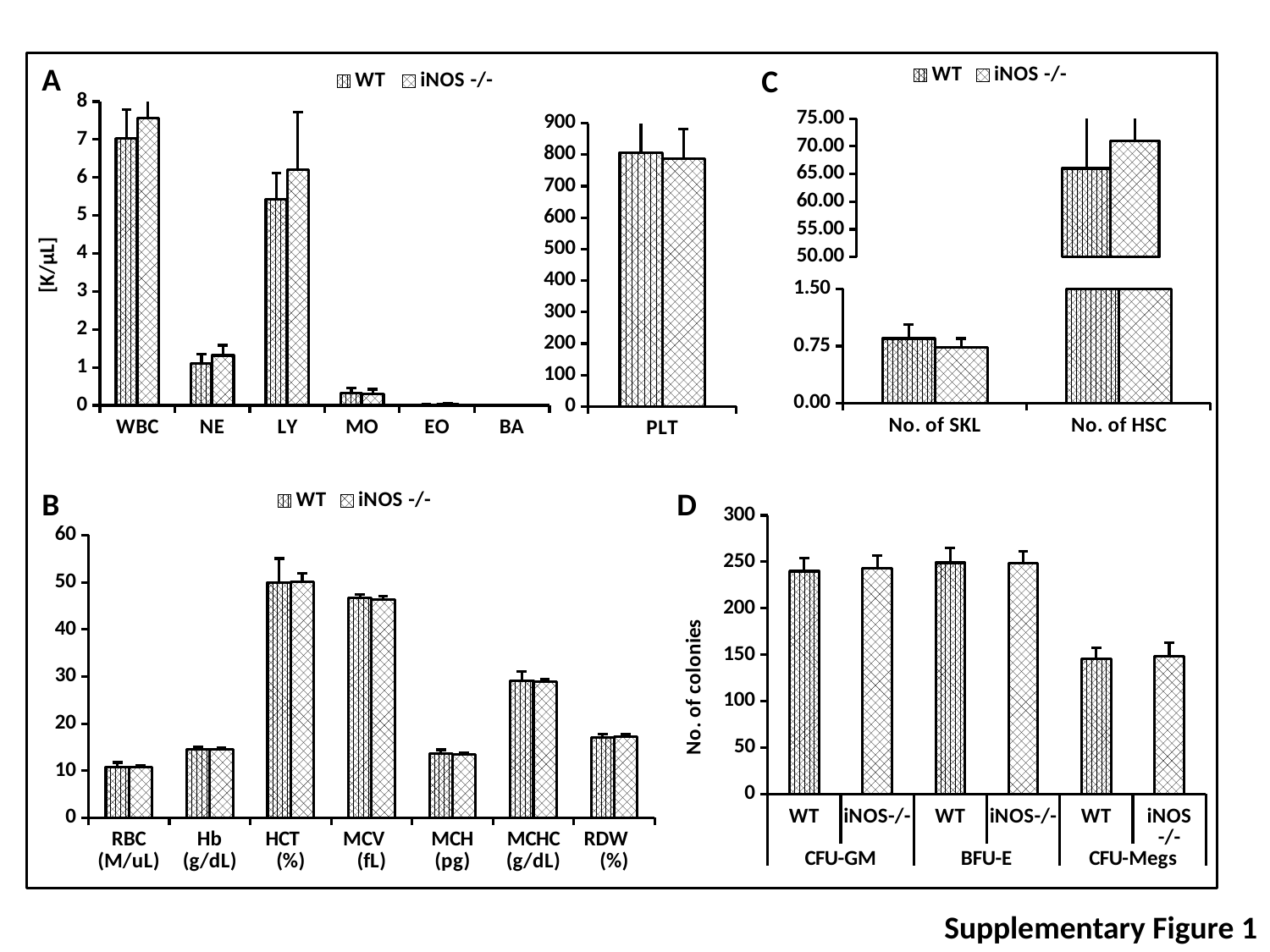

### Chart
| Category | WT | iNOS -/- |
|---|---|---|
| WBC | 7.025714285714285 | 7.571428571428571 |
| NE | 1.1099999999999997 | 1.3157142857142856 |
| LY | 5.418571428571428 | 6.205714285714286 |
| MO | 0.33000000000000007 | 0.29857142857142854 |
| EO | 0.020000000000000004 | 0.027142857142857142 |
| BA | 0.004285714285714287 | 0.007142857142857144 |
### Chart
| Category | WT | iNOS -/- |
|---|---|---|
| No. of SKL | 0.8500000000000001 | 0.7300000000000001 |
| No. of HSC | 66.0 | 71.0 |
A
C
### Chart
| Category | WT | iNOS -/- |
|---|---|---|
| PLT | 806.1428571428571 | 788.0 |
### Chart
| Category | WT | iNOS -/- |
|---|---|---|
| No. of SKL | 0.8500000000000001 | 0.7300000000000001 |
| No. of HSC | 66.0 | 71.0 |
### Chart
| Category | WT | iNOS -/- |
|---|---|---|
| RBC (M/uL) | 10.712857142857143 | 10.818571428571426 |
| Hb (g/dL) | 14.471428571428572 | 14.471428571428572 |
| HCT (%) | 49.98571428571427 | 50.12857142857144 |
| MCV (fL) | 46.64285714285715 | 46.35714285714285 |
| MCH (pg) | 13.571428571428571 | 13.414285714285715 |
| MCHC (g/dL) | 29.12857142857143 | 28.88571428571428 |
| RDW (%) | 17.08571428571428 | 17.185714285714283 |B
D
### Chart
| Category | |
|---|---|
| WT | 239.83333333333337 |
| iNOS-/- | 243.16666666666663 |
| WT | 249.0 |
| iNOS-/- | 248.66666666666663 |
| WT | 145.83333333333337 |
| iNOS -/- | 148.0 |Supplementary Figure 1
