# Supplementary figures and images for: Inducible Nitric Oxide Synthase (iNOS) Is a Novel Negative Regulator of Hematopoietic Stem/Progenitor Cell Trafficking
Source: Stem Cell Rev. 2016 Oct 17;13(1):92–103. doi: 10.1007/s12015-016-9693-1 (PMC5346113; doi:10.1007/s12015-016-9693-1)

## Slide 1
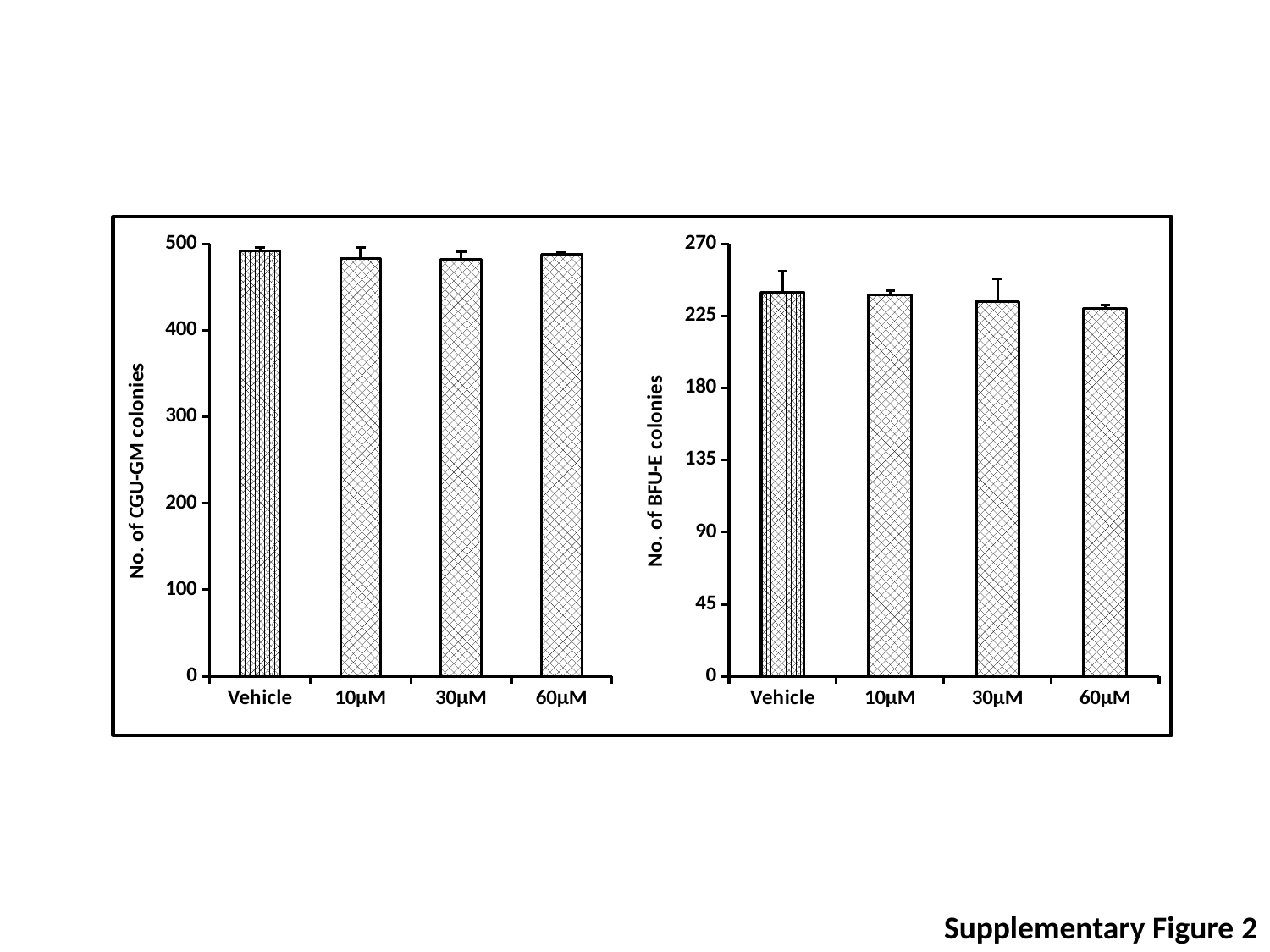

### Chart
| Category | |
|---|---|
| Vehicle | 492.0 |
| 10µM | 483.5 |
| 30µM | 482.0 |
| 60µM | 487.5 |
### Chart
| Category | |
|---|---|
| Vehicle | 239.5 |
| 10µM | 238.0 |
| 30µM | 234.0 |
| 60µM | 229.5 |Supplementary Figure 2

Supplement: Supplementary file 2 — L-NIL toxicity studies. Toxicity studies of an iNOS inhibitor (L-NIL) were performed based on evaluation of the number of CFU-GM (Panel left) and BFU-E (Panel right) clonogenic progenitors in in vitro assays. Murine bone marrow mononuclear cells were incubated with medium alone or different doses of L-NIL for 1 h and then supplemented for CFU-GM and BFU-E colonies. BM hematopoietic clonogenic progenitors were scored after 7 days of incubation, and data from two separate experiments are pooled together. (PPTX 72 kb) [file 12015_2016_9693_MOESM2_ESM.pptx]
