# Supplementary material for: Inducible Nitric Oxide Synthase (iNOS) Is a Novel Negative Regulator of Hematopoietic Stem/Progenitor Cell Trafficking
Source: Stem Cell Rev. 2016 Oct 17;13(1):92–103. doi: 10.1007/s12015-016-9693-1 (PMC5346113; doi:10.1007/s12015-016-9693-1)
Supplement: Supplementary file 3 — Expression of HO-1 directly correlates with iNOS expression in hematopoietic cell lines. Expression of iNOS in the RAJI cell line with up- and downregulation of HO-1 was analyzed by qPCR (panels A and C). In parallel, we measured the NO levels in these cells as described in the Materials and Methods section (panels B and D). *p ≤ 0.005. (PPTX 85 kb) [file 12015_2016_9693_MOESM3_ESM.pptx]

## Slide 1
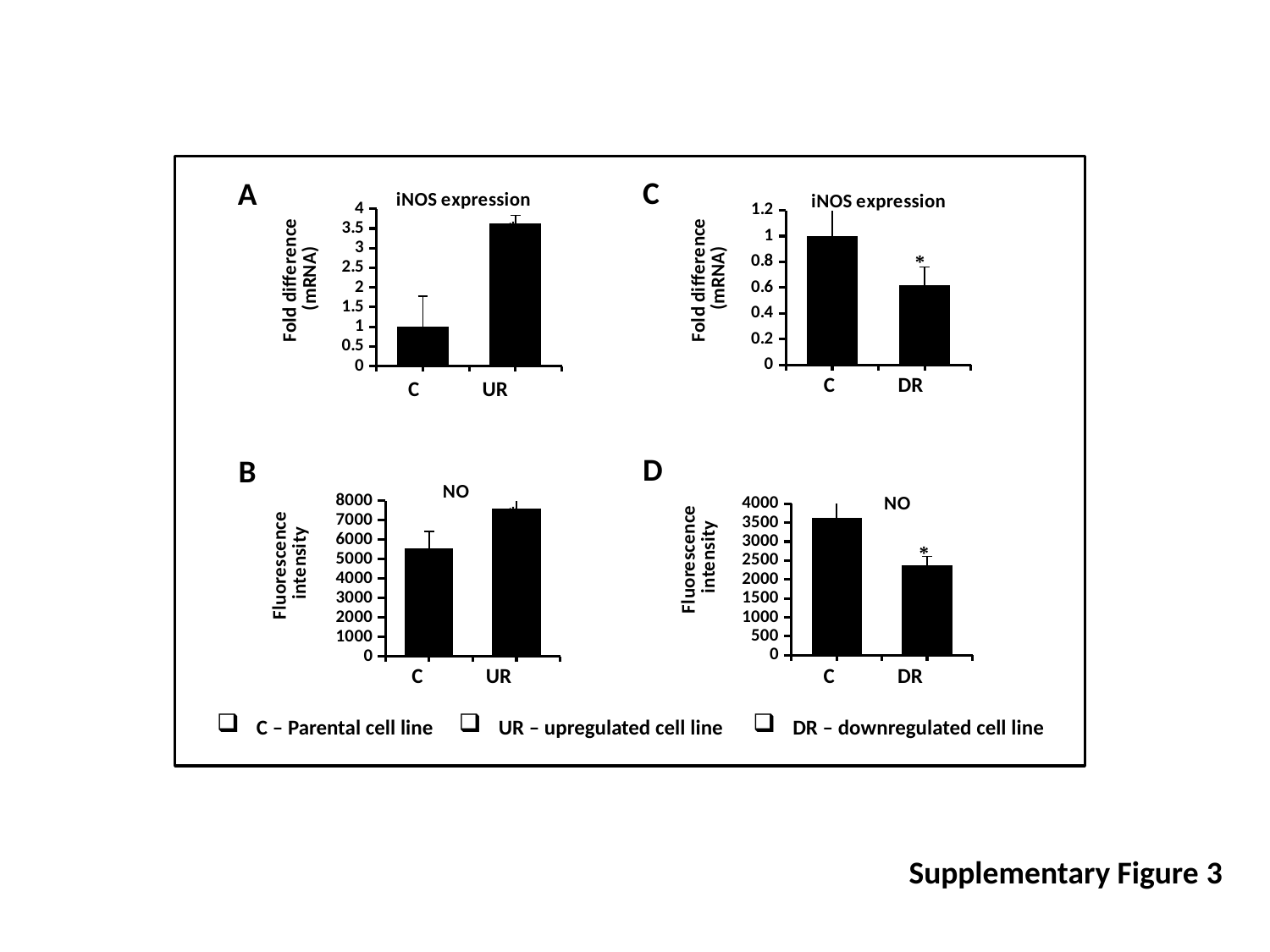

C
A
### Chart: iNOS expression
| Category | |
|---|---|
| pEGFP-C2 | 1.0 |
| pCMV6-HO-1 | 3.61751751 |
### Chart: iNOS expression
| Category | |
|---|---|
| shRNA | 1.0 |
| shRNA-HO-1 | 0.617709319 |*
*
C DR
C UR
### Chart: NO
| Category | |
|---|---|
| pEGFP-C2 | 5538.0 |
| pCMV6-HO-1 | 7577.5 |
### Chart: NO
| Category | |
|---|---|
| shRNA | 3632.0 |
| shRNA-HO-1 | 2368.5 |*
*
C UR
C DR
D
B
C – Parental cell line
UR – upregulated cell line
DR – downregulated cell line
Supplementary Figure 3
